# Bilateral Lower-Body Force Asymmetry and Countermovement Jump Performance of South African Amateur Padel Athletes

**DOI:** 10.3390/sports14100467

**Published:** 2026-10-20

**Authors:** Caeleigh King, Heather Morris-Eyton, Lynn Smith

**Affiliations:** Department of Sport and Movement Studies, Faculty of Health Science, Doornfontein Campus, University of Johannesburg, Johannesburg 2028, South Africa

**Keywords:** amateur padel, South Africa, mechanism of injury, counter movement jump

## Abstract

The countermovement jump (CMJ) is a widely used assessment of lower-limb power and neuromuscular function. The physical demands of padel, including repeated accelerations, decelerations, changes of direction, jumping, and landing, may contribute to inter-limb asymmetries influencing performance and injury risk. This quantitative cross-sectional study investigated lower-limb asymmetries during the CMJ in amateur (non-professional) padel athletes. Thirty participants (mean age: 41.2 ± 14.5 years; 63% male, 37% female) completed bilateral CMJ assessments using VALD (Brisbane, Australia) ForceDecks force plates. Outcome measures included concentric, eccentric, and landing peak force, peak power, rate of force development and jump height. Significant dominant-limb advantages were identified for concentric (*p* = 0.002), eccentric (*p* = 0.024), and landing peak force (*p* = 0.044). Male athletes demonstrated greater jump heights than female athletes (*p* < 0.001), while female athletes exhibited greater landing-phase force asymmetry (*p* = 0.004). Amateur padel athletes display significant inter-limb asymmetries in force production across the concentric, eccentric, and landing phases of the CMJ, with greater force generation in the dominant limb. Furthermore, the greater landing asymmetry observed in female athletes may reflect reduced inter-limb load distribution during landing tasks. These results highlight the value of CMJ assessment in identifying neuromuscular asymmetries that may influence performance and increase injury risk in padel athletes.

## 1. Introduction

The countermovement jump (CMJ) is a widely used assessment of lower-limb neuromuscular performance, commonly employed to evaluate force production, power, inter-limb asymmetries, fatigue, and training adaptations in athletic populations [1,2]. When performed on force plates, the CMJ provides detailed kinetic data, allowing quantification of variables such as peak force, peak power, rate of force development (RFD), and jump height [2]. Previous research has demonstrated good-to-excellent reliability of CMJ-derived metrics, including jump height, eccentric peak force, and mean force, supporting their use as valuable indicators for monitoring athletic performance and neuromuscular function [2,3].

The CMJ consists of a rapid eccentric phase followed by a concentric propulsive action and relies on the efficient utilisation of the stretch-shortening cycle (SSC) [4]. During the eccentric phase, elastic energy is stored within the musculotendinous unit and subsequently released during the concentric phase to enhance force production and movement efficiency [4]. Consequently, the CMJ has become an effective method for assessing explosive lower-limb performance in sports requiring rapid accelerations, decelerations, jumping, and changes of direction. These movement demands are characteristic of padel, a racket sport that requires repeated high-intensity multidirectional movement patterns throughout match play [5].

Padel has experienced substantial international growth; however, its physical demands pose a considerable risk of musculoskeletal injury to athletes [6,7]. The sport requires frequent accelerations, decelerations, lunges, jumps, and rapid changes of direction, which place substantial mechanical loads on the lower extremities [8]. During directional changes, loads up to 2.7 times body weight may be imposed on the ankle and knee joints, increasing tissue stress and susceptibility to injury [8]. Additional risk factors include technical errors, fatigue, inadequate physical conditioning, high training and competition loads, and repeated high-velocity movements performed during training and match play [6,8,9]. Given the frequent accelerations, decelerations, lunging, and landing actions in padel, assessing force production and absorption characteristics may provide insight into sport-specific loading strategies, monitoring athlete readiness, and identify potential neuromuscular imbalances [5].

One method of evaluating neuromuscular function is by assessing inter-limb asymmetry [2]. Inter-limb asymmetries assessed during CMJ performance may provide valuable insight into underlying movement strategies, sport-specific adaptations, and potential injury risk factors [2]. Although a degree of asymmetry is commonly observed in athletes, excessive asymmetry may reflect deficits in strength, mobility, or neuromuscular control, or a previous injury, and has been associated with altered movement mechanics and decreased performance [2]. In unilateral and asymmetrical sports, such as racket sports, repetitive loading patterns may further contribute to the development of chronic asymmetries over time, which has been proposed as a factor associated with altered movement mechanics and potential susceptibility to injury [6,7]. The CMJ offers a practical method of evaluating these factors as it allows assessment of concentric, eccentric, and landing-phase force production while simultaneously quantifying inter-limb asymmetry [2].

Most padel-related injuries occur in the lower extremities, accounting for approximately 53% of all reported injuries in both amateur and professional athletes [10,11,12]. The most frequently affected anatomical regions include the knee, calf, ankle, and hip or groin [10,11,12]. Knee injuries are of concern, with meniscal and anterior cruciate ligament (ACL) injuries commonly reported due to the sport’s multidirectional and rotational demands [10,11,12]. Previous research has also linked lower-limb injuries in padel to factors such as dynamic knee valgus, inappropriate footwear, stepping on loose balls, and repeated exposure to high mechanical loads [13]. Furthermore, the dominant limb appears to be more frequently affected, potentially owing to its primary role in force generation, propulsion, and impact absorption during sport-specific movements [8]. Playing surface characteristics may further influence injury risk. Sand-filled artificial turf courts provide increased traction and braking capacity, resulting in greater eccentric loading during rapid deceleration and directional changes [14]. Conversely, non-sand-based surfaces are generally faster and may reduce shock attenuation, potentially increasing mechanical stress on the lower-limb musculoskeletal system [14]. These factors may contribute to asymmetrical loading patterns and repetitive strain on the lower extremities throughout training and competition.

Despite the growing popularity of padel and the recognised prevalence of lower-limb injuries within the sport, little is known about the kinetic characteristics and inter-limb asymmetries of amateur padel athletes. Existing research has primarily focused on injury epidemiology, match demands, and physical fitness profiles of professional players, while investigations of limb-specific force production during functional performance tasks remain limited [9,10,11,12,13,14,15]. Consequently, the neuromuscular characteristics of amateur padel athletes, who constitute a large proportion of the playing population, remain poorly understood.

Therefore, this study aimed to investigate lower-limb force characteristics and inter-limb asymmetries during the CMJ in amateur padel athletes. Specifically, peak forces in the dominant and non-dominant limbs during the concentric, eccentric, and landing phases were compared. Secondary objectives included evaluating jump height, peak power, and RFD, as well as examining sex-related differences in CMJ-derived variables.

## 2. Materials and Methods

This study employed a quantitative, cross-sectional descriptive design. Demographic characteristics and self-reported injury data were collected using a Google Forms questionnaire administered to amateur padel, tennis, and squash athletes in Gauteng, South Africa, between January 2024 and August 2025. Participants were classified as amateur athletes if they competed exclusively at a non-professional level, including social and club-level competition.

Participants were recruited through convenience sampling from sports clubs that granted permission for data collection. The present study represents a secondary analysis of data obtained from a larger multisport investigation involving 90 amateur racket-sport athletes across padel, tennis, and squash. For the current analysis, 30 amateur padel athletes were included. Eligible participants were selected using simple random sampling and invited via email to participate in a physical assessment comprising strength and flexibility testing. Random sampling procedures were implemented to minimise selection bias and improve the representativeness of the sample.

Participants were eligible for inclusion if they were aged 18 years or older, competed in padel (as their primary sport) at an amateur level, were free from injury at the time of testing, and provided written informed consent.

### 2.1. Ethical Considerations

This research was approved by the University of Johannesburg, Faculty of Health Science, Research Ethics Committee (REC-2248-2023). All procedures were conducted in accordance with the principles of the Declaration of Helsinki. Participant anonymity, confidentiality, and informed consent were maintained throughout the research process.

### 2.2. Data Collection

Countermovement jump performance was assessed using the VALD ForceDecks dual force plate system (*ForceDecks*, VALD, Brisbane, Australia), comprising two uniaxial force plates that measure vertical GRF and sample data at 1000 Hz. Outcome variables were calculated using the manufacturer’s proprietary software. Testing was conducted in a standardised manner and required approximately 30 to 40 min per participant, with the CMJ assessment taking approximately five minutes. Participants completed three CMJs separated by a 30 s recovery period. Standardised verbal encouragement was provided throughout testing. Where necessary, additional trials were performed until three technically valid jumps had been obtained. The mean value of the three valid trials was used for analysis to improve measurement reliability and minimise trial-to-trial variability.

#### CMJ Procedure

Before testing, the force plates were calibrated according to the manufacturer’s guidelines with no load applied. Participants stood with one foot positioned on each force plate while body mass was recorded automatically. Lower-limb dominance was defined as the preferred leg for kicking a ball, consistent with previous research [15].

To standardise movement execution and minimise upper-body contribution, participants performed all jumps with their hands placed on their hips. Participants were instructed to descend to a self-selected squat depth while maintaining an upright trunk, then immediately perform a maximal vertical jump and land as softly as possible [4]. The procedure was repeated until three technically acceptable trials had been recorded.

The following CMJ-derived variables were analysed: concentric peak force, eccentric peak force, landing peak force, jump height, estimated peak power, concentric rate of force development (RFD), and eccentric RFD. Inter-limb asymmetry (%) was calculated as:

Asymmetry (%) = [(Dominant Limb − Non-Dominant Limb)/Dominant Limb] × 100

Jump height was expressed in centimetres and subsequently converted to peak power (W) using the Sayers equation [16]:

Peak Power (W) = 60.7 × (jump height [cm]) + 45.3 × (body mass [kg]) − 2055

### 2.3. Data Analysis

Data were analysed using IBM SPSS Statistics (Version 30.0; IBM Corp., Armonk, NY, USA). Descriptive statistics are presented as mean ± standard deviation (SD). Data normality was assessed using the Shapiro–Wilk test.

Differences between dominant and non-dominant limbs were evaluated using paired-samples *t*-tests. Although several variables violated the assumptions of normality, paired *t*-tests were retained as they were considered robust enough to moderate departures from normality in balanced within-subject designs. Cohen’s *d* effect sizes were calculated for paired comparisons and interpreted as small (0.20), moderate (0.50), and large (0.80).

A 2 × 2 mixed analysis of variance (ANOVA) was performed to examine the effects of limb dominance (dominant vs. non-dominant) and sex (male vs. female) on concentric, eccentric, and landing peak force. Partial eta squared (ηp^2^) was calculated as a measure of effect size and interpreted as small (0.01), medium (0.06), and large (0.14).

Multiple linear regression analysis was used to examine associations between jump height and demographic and participation-related variables. Predictors that demonstrated significant associations in preliminary analyses were retained in the final model. Statistical significance was set at *p* < 0.05.

## 3. Results

### 3.1. Participant Characteristics

Participant demographic characteristics are presented in Table 1. Thirty amateur padel athletes participated in the study, of whom 19 (63%) were male, and 11 (37%) were female. The mean age of the cohort was 41.2 ± 14.5 years, with an average height of 1.73 ± 0.09 m and body mass of 81.4 ± 14.8 kg. Most participants (60%) reported participation in tennis or squash as an additional sport.

### 3.2. Countermovement Jump Performance and Limb Comparisons

Countermovement jump performance variables are summarised in Table 2. The dominant limb exhibited significantly greater concentric peak force (*t*(29) = 3.03, *p* = 0.005, *d* = 0.55), eccentric peak force (*t*(29) = 2.60, *p* = 0.015, *d* = 0.47), and landing peak force (*t*(29) = 2.16, *p* = 0.039, *d* = 0.39) compared with the non-dominant limb. The observed effect sizes were small to moderate, with the greatest between-limb difference identified for concentric peak force.

Countermovement jump performance outcomes are presented in Table 3. The mean jump height for the total sample was 25.0 ± 9.7 cm. Male athletes demonstrated significantly greater jump heights than female athletes (29.3 ± 8.8 cm vs. 17.6 ± 6.1 cm, *p* < 0.001), accompanied by greater estimated peak power output. However, no significant sex-related differences were observed for concentric RFD (*p* = 0.196) or eccentric RFD (*p* = 0.239).

### 3.3. Inter-Limb Asymmetry (%)

Inter-limb asymmetry values for the CMJ variables are presented in Table 4. The greatest asymmetry was observed for landing peak force in female athletes, indicating larger between-limb differences during the landing phase than during the concentric or eccentric phases of the jump.

### 3.4. Effects of Limb Dominance and Sex

Results of the 2 × 2 mixed ANOVA are presented in Table 5. Significant main effects of limb dominance were observed for concentric peak force (*p* = 0.003, ηp^2^ = 0.267), eccentric peak force (*p* = 0.010, ηp^2^ = 0.216), and landing peak force (*p* = 0.031, ηp^2^ = 0.155). These effect sizes were classified as large. Significant main effects of sex were identified for eccentric peak force (*p* = 0.017, ηp^2^ = 0.188) and landing peak force (*p* = 0.004, ηp^2^ = 0.259), with males demonstrating greater force values than females. No significant sex effect was observed for concentric peak force (*p* = 0.534, ηp^2^ = 0.014). Furthermore, no significant limb × sex interactions were identified for any outcome measure (*p* > 0.05), indicating that the pattern of asymmetry was similar between males and females.

### 3.5. Predictors of Jump Height

The results of the multiple linear regression analysis are presented in Table 6 and Table 7. Following preliminary correlation analyses, sex and height were retained in the final model. The regression model was statistically significant (*F*(2, 27) = 7.363, *p* = 0.003) and explained 35.3% of the variance in jump height (*R*^2^ = 0.353). Sex emerged as the only significant predictor of jump height (β = 0.638, *p* = 0.005). Based on the unstandardised regression coefficient, male athletes achieved jump heights that were, on average, 12.67 cm greater than those of female athletes. Height was not a significant predictor of jump height (β = −0.069, *p* = 0.742).

## 4. Discussion

The present study evaluated limb-specific CMJ performance in amateur padel athletes by comparing peak force production between the dominant and non-dominant limbs across the eccentric, concentric, and landing phases.

### 4.1. Jump Height and Power

Jump height represents the vertical displacement of the body’s centre of mass and is widely used as an indicator of lower-limb power and neuromuscular performance [2,17]. The mean jump height observed in the present study (25.0 ± 9.7 cm) was lower than values reported for elite and sub-elite male padel athletes, who achieved mean jump heights of 44.6 ± 5.3 cm and 42.3 ± 6.3 cm, respectively [18].

This disparity may reflect differences in playing standard, training volume, physical conditioning, and participant characteristics, given that the current sample comprised amateur athletes. Comparisons with other sporting populations suggest that the jump heights reported in the present study are more closely aligned with those observed in recreational and team-sport athletes than with higher-level padel competitors [19,20]. Previous studies have reported median jump heights of 37.7 cm and 28.8 cm for male and female football athletes, respectively, and 39.4 cm and 28.3 cm for male and female basketball athletes, respectively [19,20].

Sex was significantly associated with jump height, with male participants demonstrating jump heights approximately 12 cm greater than those of female participants. This finding is consistent with previous literature describing sex-related differences in lower-limb power performance [12]. Greater lean muscle mass, larger muscle cross-sectional area, and higher force-producing capacity in males have been proposed as contributing factors to superior jump performance [20]. These physiological characteristics may partially explain the sex-related differences observed in the present study. However, the unequal distribution of male and female participants should be considered when interpreting these findings, as this may have influenced the magnitude of the observed differences [21].

The amateur padel athletes included in this study reported relatively low levels of sport exposure and demonstrated lower jump heights than those reported in sub-elite and professional padel populations. Male sub-elite padel athletes have been reported to achieve mean jump heights of 42.3 ± 6.2 cm, while values ranging from 32.7 ± 8.0 cm to 44.6 ± 5.3 cm have been observed in professional male padel players [18,21]. Although training exposure was not retained as a significant predictor in the regression model, these findings may suggest that athletes competing at higher standards possess greater lower-limb power and neuromuscular capabilities. It is also possible that the greater physical demands and training requirements associated with higher levels of competition contribute to these differences. Further research using longitudinal designs is required to clarify the relationship between sport exposure, training history, and jump performance in padel athletes.

Estimated peak power values in the present study were higher than those reported for elite (2257 ± 336 W) and sub-elite (2179 ± 366 W) male padel athletes [22]. However, direct comparisons should be interpreted with caution, as the participants in the referenced study were younger (27.7 ± 6.4 years) and competed at a higher performance level. Age, training history, body composition, and methodological differences may have contributed to the observed discrepancies. In contrast, the peak power values observed in the present cohort were comparable to those reported for elite handball players, suggesting that although jump height was lower than in higher-level padel athletes, peak power characteristics may be similar to those of athletes in other intermittent, high-intensity sports [3].

### 4.2. Rate of Force Development (Concentric and Eccentric)

Rate of force development (RFD) reflects the neuromuscular system’s ability to generate force rapidly and is considered an important determinant of explosive athletic performance [2]. Although RFD was quantified in the present study, previous research has highlighted concerns regarding the reliability of RFD-derived measures obtained during the countermovement jump. Specifically, Anicic et al. (2023) [2] reported coefficients of variation of 76.45% and 10.86% for concentric and eccentric RFD, respectively, suggesting considerable variability in these measures. Consequently, RFD outcomes should be interpreted cautiously, particularly when comparing individuals or drawing performance-related conclusions.

Despite these limitations, RFD remains a clinically and practically relevant measure of neuromuscular function. Improvements in RFD have been demonstrated following both explosive and heavy resistance training, with adaptations attributed to enhanced motor unit recruitment, increased firing frequency, greater musculotendinous stiffness, and improved muscle activation capacity [23]. Collectively, these adaptations facilitate more rapid force production and may contribute to improvements in explosive performance [23]. Given the dynamic and high-intensity demands of padel, training interventions aimed at enhancing rapid force-generating capacity may therefore be beneficial for athletic performance. Furthermore, maintaining or improving RFD may be particularly important across the lifespan, as age-related declines in explosive strength are often more pronounced than reductions in maximal strength [23].

### 4.3. Peak Force (Concentric, Eccentric and Landing)

The CMJ relies on effective utilisation of the stretch-shortening cycle, whereby energy stored during the eccentric phase contributes to force production in the subsequent concentric phase [5]. Eccentric peak force represents an athlete’s capacity to absorb and control loading during the braking phase and has demonstrated excellent reliability in CMJ assessments (CV = 3.34%, ICC = 0.97) [17]. The eccentric peak force values observed in the present study were lower than those reported in collegiate basketball athletes (978.0 ± 105.6 N) [24]. Similarly, concentric peak force values were lower than those reported in the same cohort of collegiate basketball athletes (1084.8 ± 58.3 N) [24]. These differences may reflect variations in sport-specific demands, training exposure, competitive level, and athlete characteristics.

Significant inter-limb differences were observed for eccentric, concentric, and landing peak force, with the dominant limb demonstrating greater force production across all phases of the CMJ. These findings suggest the presence of sport-specific neuromuscular adaptations associated with the asymmetric movement patterns characteristic of padel. Repeated unilateral loading during accelerations, decelerations, directional changes, and stroke execution may enhance force-production capabilities in the dominant limb. Given the importance of explosive movement and rapid court coverage in padel, such asymmetries may represent functional adaptations to the sport’s demands. However, excessive inter-limb asymmetry may have implications for both performance and injury risk and therefore warrants consideration during athlete monitoring [25].

Landing peak force reflects the ability to absorb and dissipate impact forces upon ground contact through coordinated lower-limb mechanics [4]. The landing peak force values observed in the present study were lower than those reported in collegiate basketball athletes (2480.7 ± 499.5 N) [24]. Because landing is associated with the largest external forces experienced during the CMJ, asymmetries occurring during this phase may provide valuable insight into limb-specific loading strategies that are not apparent during take-off [4]. Unequal force attenuation between limbs may alter movement efficiency and increase mechanical loading on specific tissues during high-impact sporting tasks. Previous research has suggested that asymmetries exceeding 15% may be associated with an elevated risk of injury, particularly during activities involving repetitive loading and landing demands [26].

The asymmetries observed in the present study were generally below the commonly cited 15% threshold, except for landing peak force in female athletes, which demonstrated an asymmetry of 18%. Although some degree of asymmetry is expected in inherently unilateral sports such as padel, larger asymmetries may indicate altered neuromuscular function, reduced load tolerance, or compensatory movement strategies [25]. Previous studies have reported that asymmetries may arise from structural differences between limbs, joint-specific deficits in strength or mobility, and residual effects of previous injuries that influence loading strategies [25]. Previous studies have demonstrated limited relationships between CMJ-derived force-time metrics and inter-limb asymmetry, with no statistically significant differences reported in some cohorts [27]. This may indicate that the neuromuscular demands of the CMJ are insufficient to elicit asymmetries that become apparent under more challenging stretch-shortening cycle conditions. Consequently, drop-jump protocols have been proposed as a more sensitive method for assessing inter-limb asymmetry because they impose greater eccentric and reactive loading demands [27].

Moreover, eccentric tasks may be particularly sensitive for detecting these deficits, as athletes often demonstrate reduced force production or compensatory movement patterns when absorbing load [25]. Consequently, assessment of eccentric peak force and associated asymmetries may provide clinically relevant information for athlete monitoring, rehabilitation, and injury-risk screening.

Significant sex-related differences were observed in eccentric and landing peak force, with males demonstrating higher values than females. These findings are consistent with established sex-related differences in muscle mass, strength, and force-generating capacity [20]. In contrast, no significant sex difference was identified for concentric peak force, suggesting that propulsive force generation may be relatively similar between male and female amateur padel athletes, despite differences in force absorption during eccentric loading and landing. Furthermore, the absence of significant limb × sex interactions indicate that the magnitude and pattern of inter-limb asymmetry were comparable between sexes. This suggests that while males and females differed in absolute force production, the asymmetric loading patterns associated with padel participation were expressed similarly in both groups.

### 4.4. Limitations

Several limitations should be considered when interpreting the findings of this study. First, all measurements were collected during a single testing session and may therefore not fully reflect participants’ habitual physical performance or account for normal day-to-day variability. In addition, a standardised warm-up protocol was not implemented before testing. As a result, differences in participant readiness, residual fatigue, or muscle stiffness may have influenced performance outcomes. Future studies should incorporate standardised warm-up procedures and repeated assessments conducted at randomised intervals to improve reliability and account for within-participant variability over time.

Injury status was determined through participant self-report, which may have introduced reporting or recall bias [28]. Participants may have underreported symptoms, inaccurately recalled previous injuries, or failed to recognise underlying musculoskeletal conditions that could have influenced performance measures [28].

The unequal distribution of male and female participants may also have influenced the observed sex-related differences and limited the generalisability of these findings across sexes. Furthermore, the relatively small sample size may have reduced the statistical power to detect smaller effects and interactions.

Finally, the cross-sectional design precludes any inference of causality between CMJ-derived variables, inter-limb asymmetries, and performance outcomes. Consequently, the observed associations should be interpreted as descriptive rather than causal. Prospective longitudinal cohort studies incorporating repeated CMJ assessments and injury surveillance are warranted to determine whether observed inter-limb asymmetries change over time and are associated with subsequent performance outcomes or injury occurrence.

## 5. Conclusions

This study investigated countermovement jump-derived force characteristics and inter-limb asymmetries in amateur padel athletes. Significant differences were identified between the dominant and non-dominant limbs in concentric, eccentric, and landing peak force, with the dominant limb consistently producing greater force. These findings indicate lower-limb asymmetries during force-production and force-absorption tasks in amateur padel athletes. Furthermore, significant sex-related differences were observed in eccentric peak force, landing peak force, and jump height, with male participants showing higher values than female participants. Female athletes also demonstrated greater landing-phase asymmetry, with landing peak force asymmetry exceeding the commonly cited 15% threshold.

Collectively, these findings suggest that CMJ assessment may be a useful tool for identifying limb-specific neuromuscular characteristics in amateur padel athletes. Monitoring force production and landing force asymmetries may help practitioners identify athletes who could benefit from targeted strength and neuromuscular training interventions to improve lower-limb function and reduce asymmetrical loading patterns.

### Recommendations

Future studies should establish sport-specific normative values for musculoskeletal asymmetries in racket sport athletes to distinguish between functional adaptations and asymmetries associated with injury risk. In addition, further investigation into countermovement jump metrics is recommended to identify variables specific to padel’s movement demands and to assess their potential utility for athlete monitoring and performance assessment.

## Figures and Tables

**Table 1:** Characteristics of participants (mean ± SD).

Variable	Total (*n* = 30)	Male (*n* = 19)	Female (*n* = 11)
Age (years)	41.2 ± 14.5	39.6 ± 13.7	41.6 ± 16.3
Height (m)	1.73 ± 0.09	1.78 ± 0.09	1.65 ± 0.05
Body mass (kg)	81.4 ± 14.8	85.8 ± 16.3	74.2 ± 11.1

**Table 2:** Limb comparisons for countermovement jump peak force variables.

Variable	Dominant Limb (N)	Non-Dominant Limb (N)	Mean Difference (N)	*p*-Value	t	Cohen’s d
Concentric Peak Force	863.1 ± 312.7	805.1 ± 254.0	57.97	0.002 *	3.026	0.55
Eccentric Peak Force	581.4 ± 157.9	550.3 ± 148.7	31.16	0.024 *	2.596	0.47
Landing Peak Force	2087.3 ± 803.3	1882.4 ± 646.6	204.84	0.044 *	2.157	0.39

* Significant at *p* < 0.05. N: Newtons.

**Table 3:** Countermovement jump performance variables by sex.

Variable	All Participants (*n* = 30)	Male (*n* = 19)	Female (*n* = 11)	*p*-Value
Jump Height (cm)	25 ± 9.7	29.3 ± 8.8	17.6 ± 6.1	<0.001 *
Peak Power (W)	3150.53	3610.3	2374.6	-
Relative Peak Power (W·kg^−1^)	38.7	42.1	32.0	-
Concentric RFD (N·s^−1^)	2282 ± 1674. 5	2586.3 ± 1960.9	1756.4 ± 854.6	0.196
Eccentric RFD (N·s^−1^)	2113.6 ± 1458.9	2355.4 ± 1727.0	1696.1 ± 707.8	0.239

* Significant at *p* < 0.05.

**Table 4:** Inter-limb asymmetry.

Variable	All Participants (%)	Male (%)	Female (%)
Concentric Peak Force	7	5	10
Eccentric Peak Force	5	3	9
Landing Peak Force	10	7	18

**Table 5:** 2 × 2 mixed ANOVA results.

Variable	Limb Effect *p* (ηp^2^)	Sex Effect *p* (ηp^2^)	Interaction *p* (ηp^2^)
Concentric Peak Force	0.003 (0.267)	0.534 (0.014)	0.316 (0.036)
Eccentric Peak Force	0.010 (0.216)	0.017 (0.188)	0.310 (0.037)
Landing Peak Force	0.031 (0.155)	0.004 (0.259)	0.447 (0.021)

**Table 6:** Multiple linear regression model predicting jump height.

Outcome Variable	R^2^	Adjusted R^2^	F	*p*-Value
Jump Height	0.353	0.305	7.363	0.003 *

* Significant at *p* < 0.05.

**Table 7:** Regression coefficients for predictors of jump height. N = 30.

Predictor	B	β	95% CI	*p*-Value
Constant	29.60	-	−44.69 to 103.89	0.421
Male Sex	12.67	0.638	4.22 to 21.12	0.005 *
Height	−7.28	−0.069	−52.08 to 37.53	0.742

* Significant at *p* < 0.05.

## Data Availability

The original contributions presented in this study are included in the article. Further inquiries can be directed to the corresponding author.

## Abbreviations

The following abbreviations are used in this manuscript:

CMJ	Countermovement jump
GRF	Ground reaction force
SSC	Stretch shortening cycle
ACL	Anterior Cruciate Ligament
RFD	Rate of force development

## References

[1] Claudino J.G., Cronin J., Mezêncio B., McMaster D.T., McGuigan M., Tricoli V., Amadio A.C., Serrão J.C. (2017). The countermovement jump to monitor neuromuscular status: A meta-analysis. J. Sci. Med. Sport.

[2] Anicic Z., Janicijevic D., Knezevic O.M., Garcia-Ramos A., Petrovic M.R., Cabarkapa D., Mirkov D.M. (2023). Assessment of Countermovement Jump: What Should We Report?. Life.

[3] Fristrup B., Krustrup P., Kristensen K.H., Rasmussen S., Aagaard P. (2024). Test–retest reliability of lower limb muscle strength, jump and sprint performance tests in elite female team handball players. Eur. J. Appl. Physiol..

[4] Chang C.-C., Chiang C.-Y. (2022). Using the Countermovement Jump Metrics to Assess Dynamic Eccentric Strength: A Preliminary Study. Int. J. Environ. Res. Public Heal..

[5] Zeni M.L.D., Nunes H.E.G., Sganzerla G., Bacciotti S.d.M. (2025). Tests in the Evaluation of Physical Performance in Racket Sports: A Scoping Review. Int. J. Sports Phys. Educ..

[6] Moreno-Pérez V., López-Samanes Á., Domínguez R., Fernández-Elías V.E., González-Frutos P., Fernández-Ruiz V., Pérez-López A., Fernández-Fernández J. (2019). Acute effects of a single tennis match on passive shoulder rotation range of motion, isometric strength and serve speed in professional tennis players. PLoS ONE.

[7] Vasenina E., Hammert W.B., Kataoka R., Dankel S.J., Buckner S.L. (2022). Injuries and Strength Training Practices in Collegiate Tennis. Sports.

[8] Martin C., Sorel A., Touzard P., Bideau B., Gaborit R., DeGroot H., Kulpa R. (2021). Influence of the forehand stance on knee biomechanics: Implications for potential injury risks in tennis players. J. Sports Sci..

[9] Pérez F., de la Rubia A., Cañadas E., Lorenzo-Calvo J., Marquina M., García-Sánchez C. (2023). Musculoskeletal injury prevalence in professional padel players. A retrospective study of the 2021 season. Phys. Ther. Sport.

[10] García-Giménez A., de la Fuente F.P., Otín C.C., Páez L.C. (2022). Performance Outcome Measures in Padel: A Scoping Review. Int. J. Environ. Res. Public Health.

[11] Demeco A., de Sire A., Marotta N., Spanò R., Lippi L., Palumbo A., Iona T., Gramigna V., Palermi S., Leigheb M. (2022). Match Analysis, Physical Training, Risk of Injury and Rehabilitation in Padel: Overview of the Literature. Int. J. Environ. Res. Public Health.

[12] Dahmen J., Emanuel K.S., Fontanellas-Fes A., Verhagen E., Kerkhoffs G.M.M.J., Pluim B.M. (2023). Incidence, prevalence and nature of injuries in padel: A systematic review. BMJ Open Sport Exerc. Med..

[13] Aguilar-Núñez D., González-Romero A., Pérez-Montilla J.J., Hamed-Hamed D., González-Muñoz A., Navarro-Ledesma S. (2026). Epidemiology, Incidence, Prevalence, and Treatment of Injuries in Padel: A Scoping Review. Appl. Sci..

[14] Rocamora-López G., Mateo-Orcajada A. (2025). Analysis of Differences in Injuries in Padel Players According to Sport-Specific Factors, Level of Physical Activity, Adherence to the Mediterranean Diet, and Psychological Status. Sports.

[15] Moreno-Pérez V., Ayala F., Fernandez-Fernandez J., Vera-Garcia F.J. (2016). Descriptive profile of hip range of motion in elite tennis players. Phys. Ther. Sport.

[16] Sayers S.P., Harackiewicz D.V., Harman E.A., Frykman P.N., Rosenstein M.T. (1999). Cross-validation of three jump power equations. Med. Sci. Sports Exerc..

[17] Heishman A., Daub B., Miller R., Brown B., Freitas E., Bemben M. (2019). Countermovement Jump Inter-Limb Asymmetries in Collegiate Basketball Players. Sports.

[18] Pradas F., Sánchez-Pay A., Muñoz D., Sánchez-Alcaraz B.J. (2021). Gender Differences in Physical Fitness Characteristics in Professional Padel Players. Int. J. Environ. Res. Public Heal..

[19] Fernandez-Fernandez J., Ulbricht A., Ferrauti A. (2014). Fitness testing of tennis players: How valuable is it?. Br. J. Sports Med..

[20] Philpott L.K., Forrester S.E., van Lopik K.A., Hayward S., Conway P.P., West A.A. (2021). Countermovement jump performance in elite male and female sprinters and high jumpers. Proc. Inst. Mech. Eng. Part P J. Sports Eng. Technol..

[21] Sánchez-Muñoz C., Muros J.J., Cañas J., Courel-Ibáñez J., Sánchez-Alcaraz B.J., Zabala M. (2020). Anthropometric and Physical Fitness Profiles of World-Class Male Padel Players. Int. J. Environ. Res. Public Health.

[22] Merrigan J.J., Stone J.D., Hornsby W.G., Hagen J.A. (2020). Identifying Reliable and Relatable Force–Time Metrics in Athletes—Considerations for the Isometric Mid-Thigh Pull and Countermovement Jump. Sports.

[23] Maffiuletti N.A., Aagaard P., Blazevich A.J., Folland J., Tillin N., Duchateau J. (2016). Rate of force development: Physiological and methodological considerations. Eur. J. Appl. Physiol..

[24] Guan Y., Bredin S., Taunton J., Jiang Q., Wu N., Warburton D. (2022). Association between Inter-Limb Asymmetries in Lower-Limb Functional Performance and Sport Injury: A Systematic Review of Prospective Cohort Studies. J. Clin. Med..

[25] McLaughlin R., Cohen D., Graham-Smith P., James L., Natera A., Kotsifaki R., Geneau M.C. (2026). Practitioner’s Advanced Guide to Force Plates. VALD|Download Free Practitioner’s Advanced Guide to Force Plates ValdperformanceCom. https://resources.valdperformance.com/practitioners-advanced-guided-to-force-plates.

[26] Natera A., Jordan M., Cohen D., James L., Howells D., French D., Clubb J. (2026). Practitioner’s Intermediate Guide to Force Plates. VALD|Download Free Practitioner’s Intermediate Guide to Force Plates ValdperformanceCom. https://resources.valdperformance.com/practitioners-intermediate-guide-to-force-plates.

[27] Kyne H., Cronin J.B. (2025). Beyond Jump Height: A Comparison of Concentric Variables in the Squat Jump, Countermovement Jump and Drop Jump for Athletic Profiling. Sports.

[28] Bong S., Lee K., Dominici F. (2024). Differential recall bias in estimating treatment effects in observational studies. Biometrics.

